# Mitral annular disjunction; how accurate are we? A cardiovascular MRI study defining risk

**DOI:** 10.1016/j.ijcha.2023.101298

**Published:** 2023-11-09

**Authors:** Nasir Hussain, Geeta Bhagia, Mark Doyle, Geetha Rayarao, Ronald B. Williams, Robert W.W. Biederman

**Affiliations:** a Allegheny Health Network, Allegheny General Hospital, Center for Cardiovascular MRI, Pittsburgh, PA 15212, USA; b Wilson Medical Center, United Health Service, Johnson City, NY 13790, USA; c Benefis Health System, Great Falls, MT 59404, USA; d West Virginia University, Morgantown, WV 26506, USA; e Medical University of South Carolina, Charleston, SC 29425, USA

**Keywords:** Mitral annular disjunction, Mitral valve prolapse, Ventricular tachycardia

## Abstract

**Aims:**

Mitral Annular Disjunction (MAD) refers to embryologic fibrous separation between mitral annular ring and basal left ventricular myocardium. Since its original description, the role of MAD in arrhythmic mitral valve prolapse (MVP) has been the subject of active research. In this study we sought to assess prognostic and imaging characteristics of MVP patients with and without underlying MAD.

**Methods and results:**

Patients with posterior or bi-leaflet MVP were retrospectively identified via a review of all patients referred to our cardiac magnetic resonance (CMR) imaging laboratory from January 2015 to May 2022. MVP patients were further stratified by underlying MAD status. A total of 100 MVP patients undergoing CMR imaging (52 MVP patients with posterior MAD) were retrospectively identified with female comprising 55 % of the cohort. MVP patients with MAD were more likely to have an abnormal basal inferolateral/ papillary muscles LGE (51 % vs 21 %, p < 0.01). Posterior MAD longitudinal disjunction gap in ‘mm’ was a predictor of ventricular tachycardia (VT) [1.29, p = 0.01)]. Using ROC curve analysis, a disjunction gap of ≥ 4 mm was predictive of VT (AUC-0.71, p < 0.01), and incorporation of LGE in ROC model further improved AUC to 0.78 confirmed via Akaike information criterion (p < 0.01).

**Conclusion:**

Abnormal LGE involving basal inferolateral myocardium and papillary muscles may provide etiologic substrate for arrythmia in MVP patients.

## 1 Introduction

Mitral valve prolapse (MVP) refers to at least 2 mm systolic displacement of the mitral leaflets into the left atrium beyond mitral annular plane measured on a parasternal long axis view of the echocardiogram [1]. MVP was first described in 1960 s using cine-angiocardiography [2]. However, using the modern definition [1], estimated prevalence of the MVP ranges anywhere from 1 % to 3 % [3], [4]. MVP is the most common cause of chronic primary mitral regurgitation (MR) with only a minority of MVP patients progressing to severe MR [5]. MVP is usually sporadic in etiology; however, genetic/ familial patterns have been described [6]. MVP may associate with other connective tissues disease such as Marfan’s, Ehlers-Danlos, and Loeys-Dietz syndrome etc. [7]. Pathologically, MVP can range anywhere from classic Barlow’s disease with thickened redundant leaflets to fibroelastic deficiency with thin leaflets [8]. The long-term outcomes of MVP patients are variable with increasing recognition of a subset that may develop ventricular arrhythmias and sudden cardiac death [3], [9].

Mitral annulus (MA) is a D-shaped fibrous ring [10]. MA posteriorly is attached to the left ventricular (LV) myocardium, during systole LV myocardium leads to sphincter like contraction of the MA allowing for proper coaptation of the mitral leaflets [10]. When there is a gap, composed of fibrosus tissue, between the posterior MA and basal LV myocardium, it is labelled as Mitral Annular Disjunction (MAD) [10]. Hence, mechanistically it is easier to imagine that MAD may lead to altered MA dynamics that may lead to development of the MVP [10]. Similarly, altered dynamics have been proposed to cause stretching and scarring of the basal inferolateral LV myocardium and the papillary muscles and thus providing substrate for ventricular arrhythmias and sudden cardiac death [11]. However, MAD has been demonstrated in structurally normal hearts as well leading to conflicting evidence regarding prognostic significance of the MAD [12].

In this paper, we explore whether presence of MAD in MVP patients as determined on cardiac magnetic resonance imaging (CMR) leads to worsening leaflet prolapse, and increased risk for arrhythmias. Further, we have also sought to determine whether presence of late gadolinium enhancement (LGE) associated with arrhythmic MVP conferring independent risk.

## 2 Methods

Patients with posterior or bi-leaflet prolapse were retrospectively identified after a thorough review of all the patients referred to our advanced CMR laboratory from January 2015 through May 2022. Patients with isolated anterior leaflet prolapse (n = 9), or isolated MAD without concurrent MVP (n = 5) were excluded. Similarly, MVP patients with pacemaker (n = 3), and or implantable cardioverter defibrillator (n = 10) at time of CMR imaging were excluded as well. Baseline clinical variables were ascertained via an IRB approved retrospective chart review.

Arrhythmia were ascertained via review of 24-hour or 7-day Holter monitoring, event monitoring, and or electrocardiogram (EKG). The presence of supraventricular tachycardia, or atrial flutter, or atrial fibrillation was labelled as an atrial arrhythmia. Similarly, presence of sustained ventricular tachycardia (VT) (lasting > 30 s) or non-sustained ventricular tachycardia (NSVT) (<30 s) was labelled as a ventricular arrhythmia. Where there was no history of palpitation with lack of subsequent EKG evidence of arrhythmia then those patients were assumed not to have atrial or ventricular arrhythmia even if Holter monitoring was not performed.

All patients underwent imaging on a 1.5 T GE (GE-CVI Excite version 12, Milwaukee, WI) CMR scanner. CMR scan protocols included steady state free precession (SSFP) 2, 3, 4-chamber views, and short-axis stack. Short-axis stacks consisted of 14–16 slices (8 mm thickness) with zero interslice gap. Three different slices of 3-Chamber views were obtained by using prescription axis that was orthogonal to each scallop to visualize A3-P3, A2-P2, and A1-P1 scallops and relationship of posterior scallops to basal LV myocardium, see Fig. 1a.

**Fig. 1a:**
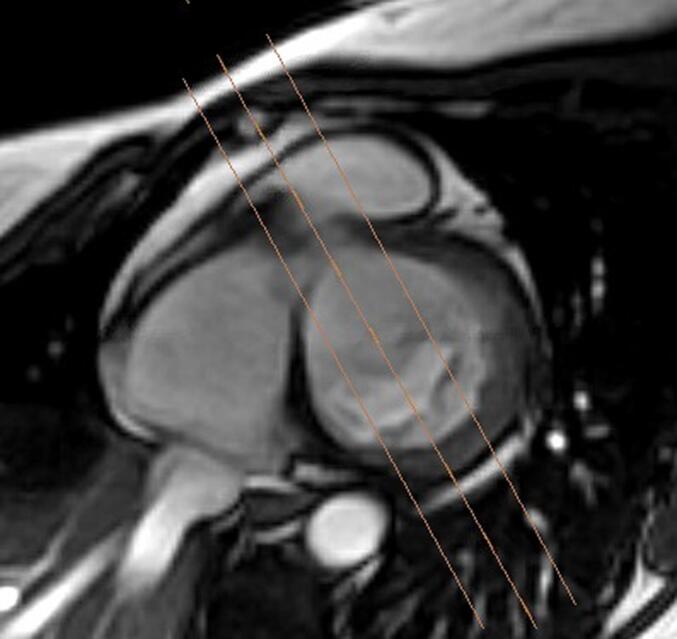
Short axis SSFP view of the left ventricle, orange lines reflect prescription axis used to obtain three different 3-chamber views at A3-P3, A2-P2, and A1-P1 locations. (For interpretation of the references to colour in this figure legend, the reader is referred to the web version of this article.)

Mitral valve leaflet systolic prolapse of at least 2 mm into the left atrium above the MA plane as determined on a 3-chamber SSFP view was used to define the MVP. Severity of prolapse for each scallop, where applicable, was determined by assessing the widest orthogonal distance of the leaflet beyond MA plane, see Fig. 1b, Fig. 1c. Posterior MAD was defined as a presence of any longitudinal disjunction gap (mm) from hinge of posterior MA to basal inferolateral LV on an end-systolic 3-chamber SSFP view, see Fig. 1c. Medial and lateral disjunctions were defined as any longitudinal gap on an end-systolic 2-chamber SSFP views, see Fig. 1d. If disjunction gap involved all three posterior scallops, medial and lateral commissures then patient was labelled to have a circumferential MAD. For anterior, and posterior MVP, widest prolapse at any scallop was considered to define the severity of the prolapse. Similarly, for posterior MAD, the widest longitudinal disjunction gap at any posterior scallop location defined the severity of the posterior MAD.

**Fig. 1b:**
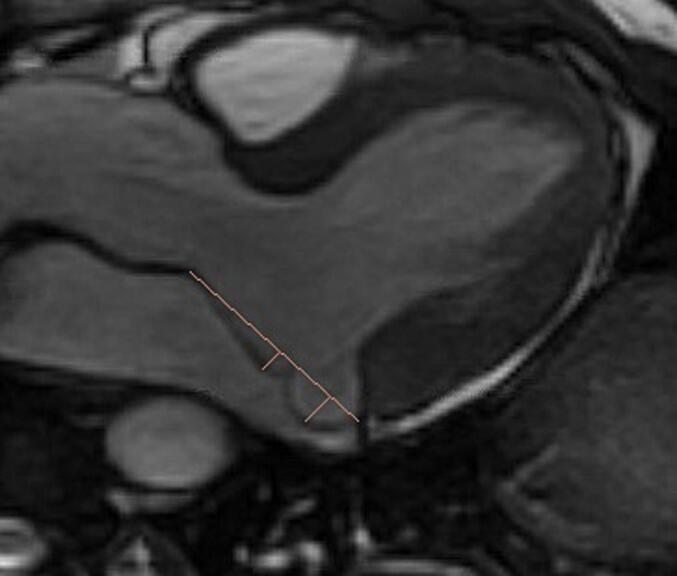
3-chamber view at A2-P2 location, orange line connecting posterior mitral leaflet hinge to posterior aortic root defines the mitral annulus area. Two orthogonal lines demonstrate method used to assess the severity of prolapse in mm.

**Fig. 1c:**
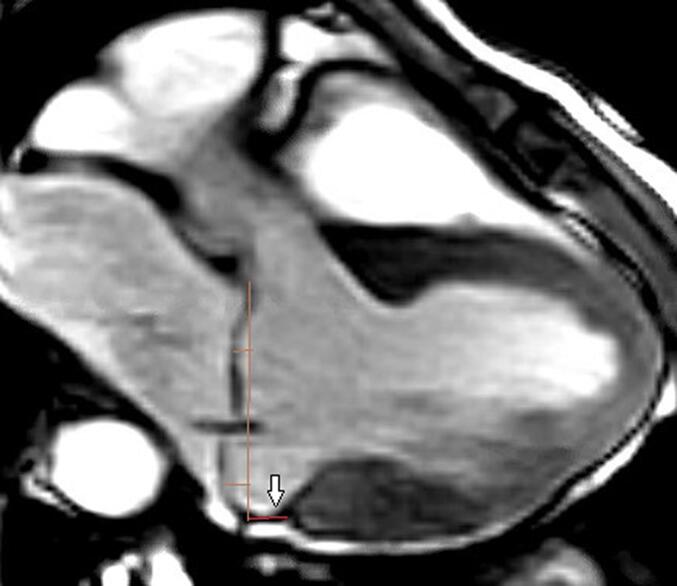
3-chamber view at A2-P2 location, arrow shows method used to assess the longitudinal posterior mitral annular disjunction gap in mm (pink line). Orange line connecting posterior mitral leaflet hinge to posterior aortic root defines the mitral annulus area. Two orthogonal lines demonstrate method used to assess the severity of prolapse in mm.

**Fig. 1d:**
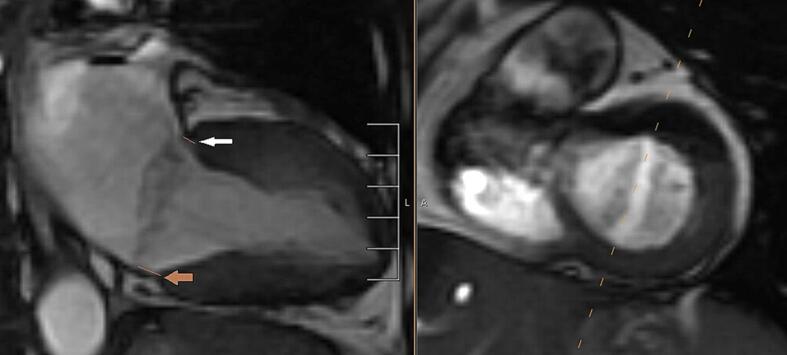
2-chamber SSFP view, note short-axis prescription used to define the 2-chamber view. Brown arrow represents medial disjunction, and white arrow represents lateral disjunction.

MA area was defined as a distance from posterior mitral leaflet hinge to posterior aortic root as determined on a 3-chamber view with prescription slice at level of A2-P2 scallops triangulating with the short-axis view for optimum dimensional analysis and reproducibility, see Fig. 1b. End-diastolic MA area, and end-systolic MA area were obtained. Delta MA area was obtained by subtracting diastolic MA area from systolic MA area. Ratio of basal inferolateral LV myocardium thickness to mid inferolateral LV wall was obtained to assess for hypertrophy involving the basal inferolateral LV segment.

**Fig. 2:**
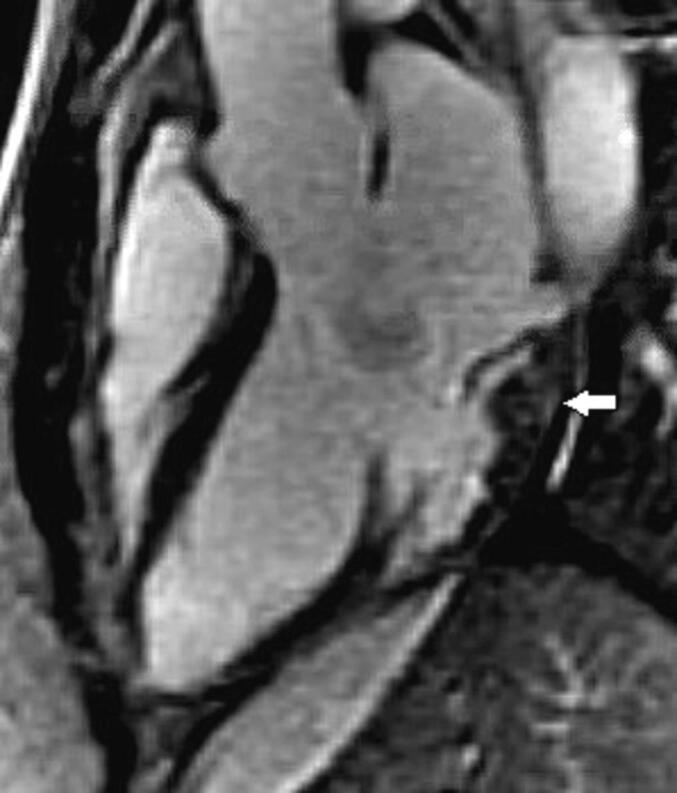
3-chamber view late gadolinium enhancement sequence, white arrow represents hyper-enhancement noted in left ventricular basal inferolateral segment reflecting presence of scar.

Late (10 min post contrast injection) LGE images were obtained using manual T1-weighted, inversion recovery preparations schemes in 2, 3, 4-chamber and short-axis views using (0.15 mmol/kg) MultiHance (Bracco Diagnostics, Princeton, NJ). Regions of myocardium with abnormally high signals (>2 standard deviations) and with manual quantification were determined to have scar. Accordingly, for the purpose of this study, LGE involving basal inferolateral LV segment and/or papillary muscles was labelled as an abnormal LGE, see Fig. 2.

Phase velocity mapping (PVM) images were obtained through the aorta and main pulmonary artery, positioned perpendicular to each vessel at approximately 3 cm above their respective valves. Medis software (Leiden, The Netherlands) was used to define endocardial and epicardial boundaries in multiple contiguous short-axis slice. These boundaries were used to calculate the chamber volumes at end-systole and at end-diastole which in turn were used to calculate the right and left ejection fractions. Mitral valve regurgitant volume was routinely obtained by subtracting aortic forward stroke volume based off the PVM from LV stroke volume obtained on volumetric data. Mitral regurgitant fraction was obtained by dividing mitral valve regurgitant volume by LV stroke volume based off volumetric data.

Categorical variables were presented as number and percentage, continuous data were analyzed as mean and standard deviation. Chi square for categorical variables, and Students-*t* test for numerical variables were used, p < 0.05 was considered significant. Univariate predictors of ventricular arrhythmia, VT, and LGE were identified using logistic regression. AUC was calculated to subsequently derive MAD threshold. Statistical analyses were performed using SPSS 18.0 (SPSS Inc., Chicago, Illinois). A Multivariate model using statistical software R was generated to assess additive role of LGE to MAD threshold in predicting VT, multiple imputation was used to accommodate for the missing data. The significance of the model was tested using the Akaike information criterion (AIC). The model with the lowest AIC was selected [13], [14]. Additional multivariate analyses, using logistic regression, for ventricular arrhythmia, VT, and LGE were analyzed using model-I containing age (years), female (%), and posterior MAD (% / mm), and model-II containing model-I with addition of basal inferolateral/ papillary muscle LGE (%).

## 3 Results

After a thorough review of n = 6,152 patients referred to our CMR laboratory from January 2015 through May 2022, a total of 100 posterior and bileaflet prolapse patients were identified. CMR images for MVP patients were reviewed in detail and all patients were stratified based on presence of inferolateral/ posterior disjunction into two groups: MVP patients with MAD (N = 52) or MVP patients without MAD (N = 48). Most common indication for CMR referral was assessment for valvular heart disease (78 %), followed by arrhythmia (16 %). Average age for study cohort was 58 ± 14 years with female comprising 55 % of total study population, see Table 1a. Average duration of follow-up for entire cohort was 2.1 ± 1.9 years.

**Table 1a:** Clinical characteristics.

	Full Population (N = 100)	Mitral Valve Prolapse without Posterior Mitral Annular Disjunction (N = 48)	Mitral Valve Prolapse with Posterior Mitral Annular Disjunction (N = 52)	p-value
Age (years)	58 ± 14	59 ± 13	56 ± 15	0.3
Female (%)	55 (55 %)	25 (52 %)	30 (58 %)	0.5
Hypertension (%)	38 (38 %)	21 (44 %)	17 (33 %)	0.2
Hyperlipidemia (%)	32 (32 %)	21 (44 %)	11 (21 %)	0.01
Diabetes (%)	6 (6 %)	5 (10 %)	1 (2 %)	0.1
Coronary Artery Disease (%)	8 (8 %)	7 (15 %)	1 (2 %)	0.02
Congestive Heart Failure (%)	15 (15 %)	7 (15 %)	8 (15 %)	1.0
Smokers (%)	26 (26 %)	11 (23 %)	15 (29 %)	0.8
Antihypertensives (%)	36 (36 %)	17 (35 %)	19 (37 %)	0.9

Presence of inferolateral/ posterior MAD not only associated with a higher prevalence of bi-leaflet prolapse (p = 0.01), but also worsening anterior leaflet prolapse (p = 0.03), in particular at the level of the A2 scallop (MVP without MAD: 0.9 ± 1.9 mm vs MVP with MAD: 2.04 ± 2.74 mm, p < 0.02), see Table 1b. Importantly, the overall severity of posterior leaflet prolapse as well as each posterior scallop prolapse did not differ between the two groups. The average inferolateral/ posterior disjunction gap was 5.7 ± 2.2 mm. Prevalence of medial and lateral disjunction between the two groups did not differ.

**Table 1b:** Imaging characteristics.

	Full Population (N = 100)	Mitral Valve Prolapse without Posterior Mitral Annular Disjunction (N = 48)	Mitral Valve Prolapse with Posterior Mitral Annular Disjunction (N = 52)	p-value
Bi-leaflet Prolapse (%)	38 (38 %)	12 (25 %)	26 (50 %)	0.01
Severity of posterior MVP (mm)	5.6 ± 2.6	5.5 ± 2.3	5.7 ± 2.9	0.61
Severity of anterior MVP (mm)	2.0 ± 2.9	1.3 ± 2.4	2.5 ± 3.2	0.03
Posterior Disjunction Gap (mm)	–	–	5.7 ± 2.2	–
Medial Disjunction (%)	84 (84 %)	38 (79 %)	46 (88 %)	0.20
Lateral Disjunction (%)	70 (70 %)	34 (71 %)	36 (69 %)	0.90
Circumferential Disjunction (%)	18 (18 %)	–	18 (35 %)	–
Medial Disjunction Gap (mm)	5.8 ± 3.6	4.9 ± 3.2	6.6 ± 3.8	0.01
Lateral Disjunction Gap (mm)	3.7 ± 2.9	3.4 ± 2.6	3.9 ± 3.2	0.40
Mitral Annulus-Diastole (mm)	34.0 ± 5.0	33.0 ± 5.0	34.0 ± 6.0	0.40
Mitral Annulus-Systole (mm)	39.0 ± 6.0	38.0 ± 5.0	40.0 ± 6.5	0.04
Delta Mitral Annulus (mm)	5.0 ± 4.4	4.2 ± 4.4	5.7 ± 4.3	0.08
LVEDD (mm)	51.0 ± 9.0	49.0 ± 10.0	52.0 ± 7.0	0.06
LVESD (mm)	31.0 ± 8.0	30.0 ± 9.0	33.0 ± 7.0	0.10
Basal to mid inferolateral wall thickness ratio (%)	1.0 ± 0.3	0.96 ± 0.3	1.03 ± 0.3	0.32
MR (%)	93 (93 %)	45 (94 %)	48 (92 %)	0.80
Moderate to Severe MR (%)	41 (41 %)	18 (38 %)	23 (44 %)	0.50
MR regurgitant volume (ml)	35.0 ± 22.0	41.0 ± 25.0	31.0 ± 18.0	0.10
MR Regurgitant Fraction (%)	32.0 ± 14.0	33.0 ± 13.0	31.0 ± 14.0	0.80
LVEF (%)	56.0 ± 12.0	57.0 ± 9.0	56.0 ± 14.0	0.60
Left atrium surface area (cm^2^)	24.0 ± 8.0	25.0 ± 8.0	23.0 ± 9.0	0.20
LGE performed (%)	70 (70 %)	33 (69 %)	37 (71 %)	0.80
Abnormal LGE (%)	26 (37 %)	7 (21 %)	19 (51 %)	0.009

MVP = Mitral valve prolapse, LVEDD = Left ventricular end-diastolic dimension, LVESD = Left ventricular end-systolic dimension, MR = Mitral regurgitation, LVEF = Left ventricular ejection fraction, LGE = Late gadolinium enhancement.

There was a significant systolic MA expansion for entire cohort (diastolic MA area: 34 ± 5 mm vs systolic MA area: 39 ± 6 mm, p < 0.001) as well as for both the groups: MVP without MAD-diastolic MA area: 33 ± 5 mm vs systolic MA area: 38 ± 5 mm, p < 0.001; MVP with MAD: diastolic MA area: 34 ± 6 mm vs systolic MA area: 40 ± 6.5 mm, p < 0.001). However, delta MA area among the two groups was not significant, see Table 1b. In total, 70 % of the cohort had LGE imaging performed, abnormal LGE was more prevalent in MVP with MAD (p < 0.01), see Table 1b.

A total of 57 patients reported symptoms of palpitations with 23 % providing a history of syncope. There was no difference in symptoms of palpitation or syncope in MVP patients with or without MAD. A total of 24 % of patients required mitral valve repair and or replacement, however, the need for mitral valve intervention was similar among both the groups. Although there was no difference in prevalence of NSVT in the 2 groups, MVP patient with MAD did have a higher occurrence of VT (19 % vs 2 %, p < 0.01), see Table 2.

**Table 2:** Outcomes by posterior disjunction status.

	Full Population (N = 100)	Mitral Valve Prolapse without Posterior Mitral Annular Disjunction (N = 48)	Mitral Valve Prolapse with Posterior Mitral Annular Disjunction (N = 52)	p-value
Syncope (%)	23 (23 %)	8 (17 %)	15 (29 %)	0.15
Ventricular Arrhythmia (%)	40 (40 %)	14 (29 %)	26 (50 %)	0.03
VT (%)	11 (11 %)	1 (2 %)	10 (19 %)	0.006
Atrial Arrhythmia (%)	42 (42 %)	22 (46 %)	20 (38 %)	0.5
Mitral valve intervention (%)	24 (24 %)	11 (23 %)	13 (25 %)	0.9

VT = sustained ventricular tachycardia.

During an average follow-up of 2.1 ± 1.9 years, 2 patients experienced VT/ VF sudden cardiac arrest and both the patients had MVP with MAD. One of these patients was successfully resuscitated (bystander CPR performed while in the aisle of a transcontinental airplane flight) while the other patient unfortunately died at age of 24 years. VT ablation was performed in 3 patients of which 2 had MVP with MAD. Preventative ICD was offered to one MVP with MAD patient with VT on Holter, however, the patient declined. During course of a follow-up, 5 patients underwent ICD implantation for progressive VT/NSVT, and all had underlying MVP with MAD.

**Fig. 3:**
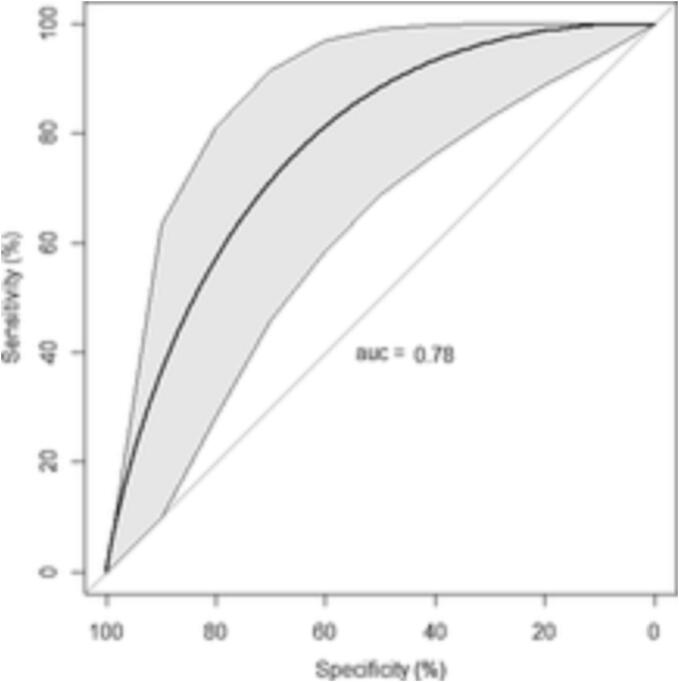
ROC plot for prediction of ventricular tachycardia (Multivariate model using disjunction gap (mm) and presence of abnormal basal inferolateral/ papillary muscles late gadolinium enhancement).

A total of 11 % of the study population experienced sustained VT whereas 40 % of the study population experienced ventricular arrhythmia. Univariate and multivariate predictors for ventricular arrhythmia, ventricular tachycardia, and LGE are presented in Table 3a, Table 3b. Via AUC of 0.71, a MAD threshold of ≥ 4 mm (p < 0.01) was identified as a significant predictor of VT. Addition of LGE to the ROC multivariate model with disjunction gap (mm) improved AUC from 0.71 to 0.78, confirmed via AIC, p < 0.01, See Fig. 3.

**Table 3a:** Univariate predictors: p-value [Odds ratio (confidence intervals)].

	Ventricular Arrhythmia	Ventricular Tachycardia	Late Gadolinium enhancement*
	p- Value	p-Value	p-Value
Age (years)	0.30	0.32	0.70
Female (%)	0.11	0.60	0.04[2.97(1.04–8.50)]
Hypertension (%)	0.80	0.95	0.80
Hyperlipidemia (%)	0.14	0.32	0.20
Diabetes (%)	0.80	0.40	0.30
Coronary Artery Disease (%)	0.12	0.90	0.10
Congestive Heart Failure (%)	0.31	0.60	0.40
Smokers (%)	0.50	0.55	0.70
Antihypertensives (%)	0.94	0.53	0.90
Bi-leaflet Prolapse (%)	0.24	0.60	0.60
Posterior/ Inferolateral Disjunction (%)	0.03[2.40(1.10–5.50)]	0.003 [11.0(1.40–91)]	0.01[3.90(1.40–11.30)]
Severity of posterior Disjunction (mm)	0.08	0.03 [1.23(1.02–1.48)]	0.04[1.20(1.01–1.40)]
Severity of posterior MVP (mm)	0.34	0.72	0.20
Severity of anterior MVP (mm)	0.30	0.50	0.90
Medial Disjunction (%)	0.06	0.50	0.80
Lateral Disjunction (%)	0.67	0.83	0.90
Circumferential Disjunction (%)	0.30	0.80	0.002[7.30(2.0–27.0)]
Medial Disjunction Gap (mm)	0.20	0.80	0.80
Lateral Disjunction Gap (mm)	0.20	0.23	0.96
Mitral Annulus-Diastole (mm)	0.31	0.80	0.70
Mitral Annulus-Systole (mm)	0.83	0.20	0.50
Delta Mitral Annulus (mm)	0.40	0.15	0.60
LVEDD (mm)	0.90	0.40	0.70
LVESD (mm)	0.91	0.20	0.80
Basal to mid inferolateral wall thickness ratio (%)	0.10	0.40	0.07
Moderate to Severe MR (%)	0.30	0.10	0.30
MR regurgitant volume (ml)	0.99	0.85	0.06
MR Regurgitant Fraction (%)	0.50	0.84	0.20
LVEF (%)	0.85	0.03 [0.93(0.88–0.99)]	0.80
Left atrium surface area (cm^2^)	0.94	0.90	0.40
Abnormal LGE (%)	<0.001[17(5–62)]	0.02 [5.3(1.2–22.6)]	–
Atrial Arrhythmia (%)	0.01[2.9(1.3–6.7)]	0.12	0.40

MVP = Mitral valve prolapse, LVEDD = Left ventricular end-diastolic dimension, LVESD = Left ventricular end-systolic dimension, MR = Mitral regurgitation, LVEF = Left ventricular ejection fraction, LGE = Late gadolinium enhancement.

*Late Gadolinium Enhancement involving basal inferolateral segment and or papillary muscles.

**Table 3b:** Multivariate [Odds ratio (confidence intervals, p-vaue)] predictors of ventricular arrhythmia, Ventricular tachycardia, and LGE (Late Gadolinium Enhancement).

	Ventricular Arrhythmia	Ventricular Tachycardia	LGE		
	Model-I	Model-II	Model-I	Model-II	Model-I
Posterior MAD (%)	2.55 (1.10–6.00, p-0.03)	1.95 (0.60–6.50, p-0.30)	12.40 (1.50–102.8, p-0.01)	7.80 (0.85–67.60, p-0.07)	3.80 (1.30–11.2, p-0.01)
Female (%)	1.80 (0.80–4.30, p-0.20)	1.64 (0.50–5.60, p-0.40)	1.20 (0.30–4.70, p-0.80)	0.99 (0.18–5.60, p-0.99)	2.84 (0.95–8.50, p-0.06)
Age (years)	1.02 (0.99–1.10, p-0.30)	0.99 (0.93–1.04, p-0.60)	1.03 (0.98–1.10, p-0.20)	1.00 (0.95–1.07, p-0.85)	1.00 (0.96–1.04, p-0.94)
LGE (%)	–	15.10 (4.04–56.40, p < 0.0001)	–	3.30 (0.64–17.10, p-0.15)	–
Posterior MAD (mm)	1.14(1.01–1.30, p-0.04)	1.09(0.91–1.30, p-0.40)	1.29(1.05–1.60, p-0.01)	1.22 (0.97–1.50, p-0.08)	1.19(1.01–1.40, p-0.03)
Female (%)	2.00 (0.90–4.80, p-0.10)	1.80 (0.50–6.20, p-0.40)	1.70 (0.40–6.70, p-0.50)	1.40 (0.25–7.20, p-0.71)	3.40 (1.09–10.40, p-0.03)
Age (years)	1.02 (0.99–1.10, p-0.20)	0.99 (0.93–1.04, p-0.60)	1.03 (0.98–1.10, p-0.20)	1.00 (0.94–1.07, p-0.84)	1.00 (0.96–1.05, p-0.96)
LGE (%)	–	15.70 (4.20–57.00, p < 0.0001)	–	3.70 (0.74–18.10, p-0.10)	–

Model-I contains the following variables: Age (years), female (%), and posterior MAD (Mitral Annular Disjunction) (%)/ mm.

Model-II contains the following variables: Age (years), female (%), posterior Mitral Annular Disjunction (%)/ mm, and basal inferolateral/ papillary muscles LGE (%).

Mitral valve repair/ replacement univariate predictors are provided in supplemental table-1. However, the presence of MAD and LGE did not predict subsequent need for mitral valve intervention. Imaging and clinical characteristics of the study population by basal inferolateral/ papillary muscles LGE status are presented in supplemental Table 2.

## 4 Discussion

There are three significant findings in this study. Posterior MAD (longitudinal disjunction gap measured in mm) even after adjustment for age, and gender was associated with the VT. Each increase of one mm in posterior disjunction gap conferred 29 % increase in odds ratio of having VT. The second finding was the establishment, for the first time, of a MAD threshold for prediction of VT. We have identified for the first time that a CMR-defined threshold ≥ 4 mm MAD (AUC-0.71) in any of the posterior scallops retrospectively predicted the highest risk cohort (73 % (8 out of 11)) of VT events p < 0.01. Third, addition of the LGE in the ROC model together with MAD distance (mm) improved AUC from 0.71 to 0.78 for prediction of VT (p < 0.01), indicating LGE may have additive role in identifying high risk patients with MAD.

In a study by Dejgaard *et al*. of 116 patients with MAD where 83 patients were confirmed to have MAD by CMR (MAD with MVP (N = 80), MAD without MVP (N = 3)), prevalence of VT was 12 % [15]. LGE sequence was reported in 58 % (N = 68) of their cohort, prevalence of abnormal LGE (univariate analysis) associated with VT (7.4, p = 0.04) [15]. In our study LGE sequence was performed in 70 % (N = 70) of the study population and 71 % (N = 37) in MVP with MAD group. While a total of 11 patients (11 %) experienced sustained VT, 10 of these had underlying MVP with MAD (19 %). Abnormal LGE associated with the VT, ventricular arrhythmia in entire population, see Table 3a, Table 3b. Similarly, in our subgroup analysis of MVP with MAD patients with respect to LGE status demonstrated higher occurrence of ventricular arrhythmia (OR-18, p < 0.001) in LGE positive patients. Similarly, multivariate model containing MAD disjunction gap (mm) and positive LGE provided better AUC at 0.78 for VT outcome (p < 0.01). Overall, these findings pathophysiologically support an abnormal LGE involving inferolateral LV/ papillary muscles does confer higher risk for ventricular arrhythmia as shown by others [16], [17], [18]. Further, Dejgaard *et al*. demonstrated that LVEF associated with VT (0.86, p < 0.01) [15], our findings for total population were similar [0.93, p = 0.03). Although the study by Dejgaard *et al*. had more MVP with MAD patients (N = 80 by CMR as opposed to 52 in our study), however, an important distinction is their study did *not* have a comparator group of MVP without MAD (N = 48 in our cohort).

Our study supports the findings of altered dynamics involving the MA in patients with MVP. We found that both in MVP with MAD or MVP without MAD, there is systolic expansion involving the MA and these findings confirm previously reported literature [11]. Further, we found that systolic expansion of MA did not significantly change with presence of posterior MAD. Altered dynamics have been shown to alter the annulus tension [19] and this has been proposed to cause stretching of inferolateral wall leading to scar formation involving inferolateral wall and papillary muscles [11]. Unlike study by Marra *et al*., we did not find any consistent evidence of basal inferolateral LV hypertrophy in either group [11].

The presence of MAD has been shown to associate not only with bi-leaflet prolapse but also worsening severity of the leaflet prolapse [20]. In our study, MVP patients with MAD did have a higher prevalence of the bi-leaflet prolapse (50 % vs 25 % in the other group, p = 0.01) and worsening severity of the anterior leaflet prolapse (p = 0.03). These findings are consistent with what has been reported by others [20].

Various authors have demonstrated that the presence of posterior MAD is associated with a higher risk for mitral valve interventions in patients with connective tissue diseases such as Marfan’s syndrome and Loeys-Dietz syndrome [21], [22]. However, the presence of posterior disjunction was not associated with the need for mitral valve intervention in our study. One patient in our cohort had Marfan’s syndrome and one patient had Loeys-Dietz syndrome. Interestingly, posterior MAD was not associated with a higher risk of syncope in our study.

Recently, there has been literature published by our group [23] and others on tricuspid annular disjunction and its association with the MAD [24], however, we did not have dedicated RV inflow views available in our population, therefore, data on tricuspid annular disjunction was not formally obtained.

**Fig. 4:**
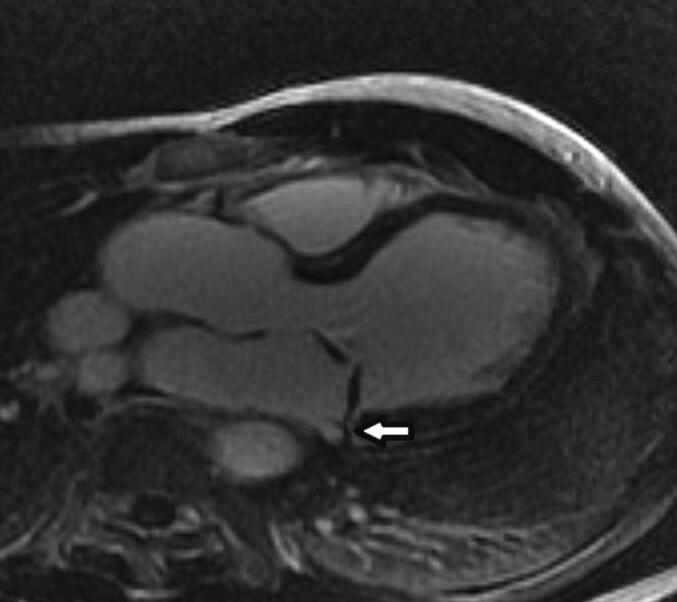
3-chamber view late gadolinium enhancement sequence, white arrow shows blood pool below mitral annulus and above basal inferolateral left ventricular myocardium.

Finally, in our experience sometimes identifying subtle posterior disjunction gap could be difficult even on CMR images due to various artifacts, and accordingly, we have found blood pool on contrast enhanced imaging may nicely help delineate disjunction gap, see Fig. 4.

## 5 Conclusion

We have identified that a disjunction gap ≥ 4 mm predicts VT (AUC-0.71), and presence of LGE adds to this risk (AUC-0.78). Here we propose that CMR may play an important role in identifying high risk features for arrhythmogenic MVP-MAD patients.

## 6 Limitations

There are several limitations involving this study. First, retrospective study design prohibits us from assessing certain imaging characteristics of our population. However, in general, we had a comprehensive valve CMR protocol performed for most of our study patients that did provide us with robust imaging data. Second, LGE imaging was performed in 70 % of the cohort only. Decision for performing LGE was clinically determined at the time of the CMR scan. Data involving only basal inferolateral/ papillary muscle LGE was collected for purpose of this study. Third, Holter monitoring was performed in 61 % of the total population, although EKGs were available for 100 % of the cohort. Fourth, for determination of ventricular or atrial arrhythmia, patients without any history of palpitations and with normal EKG were assumed not to have ventricular or atrial arrhythmia thus introducing some bias. However, clinically silent VT is a rare phenomenon, therefore, we feel all VT events or at least hemodynamically significant events were captured. Fifth, despite of this being the largest CMR study comparing MVP with and without MAD cohorts, the population sample is relatively small in this under-recognized cohort and VT event rates are lower. Additionally, the mean follow-up is approximately 2 years potentially under-estimating arrhythmia occurrences. Finally, posterior, or bi-leaflet MVP patients were diagnosed by 5 different Level III trained CMR specialists with varied clinical experience and were confirmed by first author (Level III in CMR) with kappa coefficient of 0.99. The kappa coefficient for MAD was 0.96 (first & last author).

## Declaration of Competing Interest

The authors declare that they have no known competing financial interests or personal relationships that could have appeared to influence the work reported in this paper.
