# Supplementary material for: Mitral annular disjunction; how accurate are we? A cardiovascular MRI study defining risk
Source: Int J Cardiol Heart Vasc. 2023 Nov 9;49:101298. doi: 10.1016/j.ijcha.2023.101298 (PMC10682655; doi:10.1016/j.ijcha.2023.101298)
Supplement: Supplementary data 1 [file mmc1.docx]

|  | Mitral Valve intervention  (N=24) | |
| --- | --- | --- |
|  | p- Value |  |
| Age (years) | 0.20 |  |
| Female (%) | 0.53 |  |
| Hypertension (%) | 0.23 |  |
| Hyperlipidemia (%) | 0.90 |  |
| Diabetes (%) | 0.20 |  |
| Coronary Artery Disease (%) | 0.90 |  |
| Congestive Heart Failure (%) | 0.44 |  |
| Smokers (%) | 0.90 |  |
| Antihypertensives (%) | 0.40 |  |
| Bi-leaflet Prolapse (%) | 0.70 |  |
| Posterior/ Inferolateral Disjunction (%) | 0.82 [1.12(0.44-2.81)] |  |
| Severity of posterior Disjunction (mm) | 0.42 [1.06(0.92-1.22)] |  |
| Severity of posterior MVP (mm) | 0.004 [1.30(1.10-1.60)] |  |
| Severity of anterior MVP (mm) | 0.20 |  |
| Medial Disjunction (%) | 0.60 |  |
| Lateral Disjunction (%) | 0.55 |  |
| Circumferential Disjunction (%) | 0.70 |  |
| Medial Disjunction Gap (mm) | 0.80 |  |
| Lateral Disjunction Gap (mm) | 0.92 |  |
| Mitral Annulus-Diastole (mm) | 0.005 [1.14(1.04-1.25)] |  |
| Mitral Annulus-Systole (mm) | 0.002 [1.13(1.04-1.23)] |  |
| Delta Mitral Annulus (mm) | 0.31 |  |
| LVEDD (mm) | 0.03 [1.07(1.01-1.15)] |  |
| LVESD (mm) | 0.30 |  |
| Basal to mid inferolateral wall thickness ratio (%) | 0.74 |  |
| Moderate to Severe MR (%) | <0.001 [20(5.3-73.0)] |  |
| MR regurgitant volume (ml) | 0.003 [1.05(1.02-1.08)] |  |
| MR Regurgitant Fraction (%) | 0.001 [1.10(1.04-1.17)] |  |
| LVEF (%) | 0.22 |  |
| Left atrium surface area (Cm^2^) | 0.002 [1.11(1.04-1.20)] |  |
| Abnormal LGE (%) | 0.1 |  |
| Atrial Arrhythmia (%) | 0.60 |  |
| LV mass index (g/m2) | 0.02 [1.04(1.01-1.07)] |  |
| LV end diastolic volume index (ml/m2) | 0.05 |  |
| LV stroke volume index (ml/m2) | <0.001 [1.07(1.03-1.11)] |  |

Supplemental Table I: Univariate predictors of mitral valve interventions: p-value [Odds ratio (confidence intervals)].

MVP=Mitral valve prolapse, LVEDD=Left ventricular end diastolic dimension, LVESD=Left ventricular end systolic dimension, MR=Mitral regurgitation, LVEF=left ventricular ejection fraction, LGE=Late gadolinium enhancement, LV=Left ventricle
