# Supplementary material for: Mitral annular disjunction; how accurate are we? A cardiovascular MRI study defining risk
Source: Int J Cardiol Heart Vasc. 2023 Nov 9;49:101298. doi: 10.1016/j.ijcha.2023.101298 (PMC10682655; doi:10.1016/j.ijcha.2023.101298)
Supplement: Supplementary data 2 [file mmc2.docx]

|  | Full Population | Late Gadolinium Enhancement | | p-value |
| --- | --- | --- | --- | --- |
|  |  | Negative | Positive |  |
|  | N=70 | N=44 | N=26 |  |
| Age (years) | 58.9 ± 11.9 | 58.5 ± 11.8 | 59.5 ± 12.4 | 0.70 |
| Female (%) | 41 (59%) | 22 (50%) | 19 (73%) | 0.04 |
| Hypertension (%) | 27 (39%) | 18 (41%) | 9 (35%) | 0.80 |
| Hyperlipidemia (%) | 22 (31%) | 17 (3%) | 5 (18%) | 0.20 |
| Diabetes (%) | 6 (9%) | 5 (11%) | 1 (4%) | 0.30 |
| Coronary Artery Disease (%) | 6 (9%) | 5 (11%) | 1 (4%) | 0.10 |
| Congestive Heart Failure (%) | 13 (19%) | 11(25%) | 2 (8%) | 0.40 |
| Smokers (%) | 17 (24%) | 7(16%) | 10 (38%) | 0.70 |
| Antihypertensives (%) | 25 (36%) | 16 (36%) | 9 (35%) | 0.90 |
| Bi-leaflet Prolapse (%) | 25 (36%) | 15 (34%) | 10 (38%) | 0.60 |
| Posterior MAD (%) | 38 (54%) | 19 (41%) | 19 (73.1%) | 0.01 |
| Severity of posterior MVP (mm) | 5.5 ± 2.6 | 5.8 ± 2.8 | 5.0 ± 2.3 | 0.20 |
| Severity of anterior MVP (mm) | 1.8 ± 2.8 | 1.8 ± 2.9 | 1.8 ± 2.7 | 0.90 |
| Posterior Disjunction Gap (mm) | 3.0 ± 3.3 | 2.4 ± 3.2 | 4.1 ± 3.1 | 0.04 |
| Medial Disjunction (%) | 62 (88%) | 40 (91%) | 22 (85%) | 0.80 |
| Lateral Disjunction (%) | 53 (76%) | 34 (77%) | 19 (73%) | 0.90 |
| Circumferential Disjunction (%) | 15 (21%) | 4 (9%) | 11 (58%) | 0.002 |
| Medial Disjunction Gap (mm) | 5.8 ± 3.3 | 5.9 ± 3.5 | 5.6 ± 3.1 | 0.80 |
| Lateral Disjunction Gap (mm) | 3.9 ± 2.9 | 3.9 ± 2.9 | 3.8 ± 3.0 | 0.96 |
| Mitral Annulus-Diastole (mm) | 34.0 ± 5.3 | 34.0 ± 5.7 | 34.0 ± 4.7 | 0.70 |
| Mitral Annulus-Systole (mm) | 39.0 ± 6.0 | 39.0 ± 6.5 | 38.0 ± 5.0 | 0.50 |
| Delta Mitral Annulus (mm) | 4.6 ± 4.3 | 4.8 ± 4.3 | 4.2 ± 4.3 | 0.60 |
| LVEDD (mm) | 50.0 ± 9.1 | 50.0 ± 8.2 | 49.0 ± 10.5 | 0.70 |
| LVESD (mm) | 31.0 ± 9.0 | 31.0 ± 8.6 | 31.0 ± 9.0 | 0.80 |
| Basal to mid inferolateral wall thickness ratio (%) | 1.0 ± 0.3 | 0.95 ± 0.24 | 1.1 ± 0.4 | 0.07 |
| Moderate to Severe MR (%) | 22 (31%) | 16 (36%) | 6 (23%) | 0.30 |
| MR regurgitant volume (ml) | 33.0 ± 22.0 | 38.0 ± 16.5 | 25.0 ± 8.0 | 0.06 |
| MR Regurgitant Fraction (%) | 31.0 ± 14.9 | 33.0 ± 16.5 | 25.0 ± 8.1 | 0.20 |
| LVEF (%) | 55.1 ± 13.5 | 55.0 ± 10.0 | 56.0 ± 18.0 | 0.80 |
| Left atrium surface area (cm^2^) | 24.0 ± 8.0 | 24.0 ± 8.0 | 23.0 ± 8.0 | 0.40 |

Supplemental Table II

MVP=Mitral valve prolapse, LVEDD=Left ventricular end diastolic dimension, LVESD=Left ventricular end systolic dimension, MR=Mitral regurgitation, LVEF=left ventricular ejection fraction.
